# Smart Homes as Enablers for Depression Pre-Diagnosis Using PHQ-9 on HMI through Fuzzy Logic Decision System

**DOI:** 10.3390/s21237864

**Published:** 2021-11-26

**Authors:** Juana Isabel Méndez, Ana Victoria Meza-Sánchez, Pedro Ponce, Troy McDaniel, Therese Peffer, Alan Meier, Arturo Molina

**Affiliations:** 1 School of Engineering and Sciences, Tecnologico de Monterrey, Mexico City 14380, Mexico; A01165549@itesm.mx (J.I.M.); armolina@tec.mx (A.M.); 2 Escuela Superior de Medicina, Instituto Politecnico Nacional, Mexico City 11340, Mexico; mezaana.med@gmail.com; 3 The Polytechnic School, Arizona State University, Mesa, AZ 85212, USA; troy.mcdaniel@asu.edu; 4 Institute for Energy and Environment, University of California, Berkeley, CA 94720, USA; tpeffer@berkeley.edu; 5 Energy and Efficiency Institute, University of California, Davis, CA 95616, USA; akmeier@ucdavis.edu

**Keywords:** smart home, PHQ-9, depression pre-diagnosis, HMI

## Abstract

Depression is a common mental illness characterized by sadness, lack of interest, or pleasure. According to the DSM-5, there are nine symptoms, from which an individual must present 4 or 5 in the last two weeks to fulfill the diagnosis criteria of depression. Nevertheless, the common methods that health care professionals use to assess and monitor depression symptoms are face-to-face questionnaires leading to time-consuming or expensive methods. On the other hand, smart homes can monitor householders’ health through smart devices such as smartphones, wearables, cameras, or voice assistants connected to the home. Although the depression disorders at smart homes are commonly oriented to the senior sector, depression affects all of us. Therefore, even though an expert needs to diagnose the depression disorder, questionnaires as the PHQ-9 help spot any depressive symptomatology as a pre-diagnosis. Thus, this paper proposes a three-step framework; the first step assesses the nine questions to the end-user through ALEXA or a gamified HMI. Then, a fuzzy logic decision system considers three actions based on the nine responses. Finally, the last step considers these three actions: continue monitoring through Alexa and the HMI, suggest specialist referral, and mandatory specialist referral.

## 1. Introduction

According to the World Health Organization, depression is a common mental disease around the world. About 5% of depressed individuals are adults, and 5.7% are senior people [1]. Besides, depression increased by 60% during Coronavirus disease (COVID-19) [2]. Recurrent and moderate to severe intensity depression have serious health conditions and can lead to suicide [1]. Young adults and people older than 60 years may be the most vulnerable group [1,2].

The Patient Health Questionnaire 9 (PHQ-9) is a short, self-administered questionnaire and a mental health depression screener based on the Diagnostic and Statistical Manual of Mental Disorders, fifth edition (DSM-5) [3,4]. Nevertheless, the common methods that health care professionals use to assess and monitor depression symptoms are face-to-face questionnaires leading to time-consuming or expensive methods [5]. Thus, efforts require taking advantage of what is accessible to the end-user to provide information to the health care professional. Proposals include using a smartphone and smartwatch to classify depression, physical activity, mood, social activity, sleep, and food intake [5] or the use of a smartphone to assess the PHQ-9 [6].

Smartphones or wearables, like smartwatches, are often used by single individuals as an extension of their life; they are devices that can help detect and monitor depressive symptoms [5,7,8]. On the other hand, smart homes have been arising due to the Internet of Things; for instance, modern household appliances have increased the comfort level of householders [9,10,11,12]. Chatbots are used to track mood and help users deal with depression [13,14]; thus, voice assistants as Siri or Alexa have become appealing for individuals. It has been reported that end-users prefer to interact with technology rather than health professionals [15].

COVID-19 impacted daily life and increased anxiety, depression, and suicide because individuals were clustered at homes or as a secondary effect of economic disruption [16]. In addition, their restrictions included zero interaction with other users by closing schools, universities, recreational facilities, or workplaces [2,17,18]. As a response, technologies at smart homes accelerated due to their necessity for daily activities such as work, study, or socializing [19]. Moreover, smart homes can help the health care sector by monitoring householders’ health through smartphones, wearables, cameras, or voice assistants connected to the home [10,20,21,22,23].

The structure of this paper is as follows: Section 2 presents the literature review regarding depression, the PHQ-9 questionnaire, and the smart homes as enablers for depression pre-diagnosis. Section 3 presents the three-step methodology used for this paper. Section 4 describes the proposed framework by step. Section 5 shows the proposed framework results and its linkage with gamified HMIs oriented for depression pre-diagnosis at home. Section 6 discusses the proposal with its advantages and limitations. Finally, Section 7 details the conclusions and future work.

## 2. Literature Review

Depression is a common mental illness characterized by sadness, lack of interest, or pleasure. It may also be accompanied by somatic characteristics such as disturbed sleep and appetite, tiredness, and poor concentration. Its effects on a patient’s physical, mental, and social well-being can lead to disability and cause a huge burden for healthcare systems and society. According to the World Health Organization (WHO), this illness affects approximately 3.8% of the 280 million people [1].

Consequently, depression is not exclusive to the older population; it can happen at any age leading to poor quality of life (QoL) and affecting the economically active population, as reported by Müller et al. [24]. They found that the self-reported mental distress, including depression and anxiety, was highly related to the days of incapacity to work through questionnaires and scales. Furthermore, this research reported that depressive patients presented 27 times more specific days of incapacity to work than healthy workers, leading to an economic burden of the disease due to its management costs.

According to the DSM-5, the diagnosis considers symptoms, behavioral changes, and the effect of these factors in someone’s life. Hence, there are nine symptoms: sleep disturbance, interest/pleasure reduction, guilt feelings or thoughts of worthlessness, energy changes, concentration impairment, appetite or weight changes, psychomotor disturbances, suicidal thoughts, and depressed mood. In addition, an individual must present 4 or 5 (one of them being depressed mood or loss of interest or pleasure) in the last two weeks to fulfill the diagnosis criteria [25].

A specialist must perform the diagnosis based on the criteria through a guided interview. However, many questionnaires and scales have been developed due to the importance of identifying early cases; these cannot diagnose the patient. However, they can spot depressive symptoms and be used as screening or pre-diagnosis methods. In addition, suicidal thoughts are an important factor to consider; they are assessed in interviews and questionnaires. Based on the WHO reports, 700,000 people die due to suicide each year, therefore, the importance of its prompt diagnosis and management [1].

### 2.1. Diagnostic and Statistical Manual of Mental Disorders, Fifth Edition (DSM-5)

This manual has been used as the base for mental diseases since 1952 when its first edition was released [26]. It helps to standardize diagnosis for research, treatment, and prognosis across different physicians, hospitals, and countries. For its development, more than 160 researchers and physicians were gathered to reach a consensus for the criteria of the different disorders based on the most updated information and studies [27]. However, this manual does not include information about treatment or management. It can only be used for diagnosis, prognosis, and assessment of treatment effectiveness [27].

### 2.2. Patient Health Questionnaire 9 (PHQ-9)

The PHQ-9 consists of 9 Likert-type questions (0 to 3). The total punctuation ranges from 0 to 27, and it allows to classify the symptomatology in no depression (0–4), mild depression (5–9), moderate depression (10–14), moderately severe depression (15–19), and severe depression (20–27). This tool was developed in 1999 by Robert Spitzer, Janet Williams, and Kurt Kroenke, and since then, it has been translated and validated in many languages and countries. The items in the questionnaire are listed below (Table 1). The shaded section means that if there are at least four or more selected answers thus, there is an existence of a depressive disorder.

Furthermore, this screening tool includes an item to assess the effect of the symptoms in the individual’s QoL and relationship with others and an item assessing suicidal tendencies. The suicidal item or item nine must be considered an important factor for management and possible hospitalization of the patient [25,28]. The approximated application time is 5 min, it is usually self-administered, but it could also be administered through an interview in person or by telephone [28].

The questionnaire has been translated and validated in different languages and countries. Most of them have found it with adequate internal consistency, reliability, and excellent correlation with other scales such as the Beck Depression Inventory. For example, in the meta-analysis by He et al., considering a cut-off point of 10, they reported a pooled sensitivity of 0.88 and specificity of 0.86, compared with the structured and semi-structured interviews for diagnosis of depression [29]. The 2019 study by McCord and Provost in a university in the US reported the internal consistency of this tool with Cronbach’s Alpha of 0.76 [30].

In June 2021, National Health and Nutrition Examination Survey (NHANES) program released a dataset corresponding to the 2017–2018 cycle and from 2019 to March 2020 answers of the PHQ-9 [31,32].

### 2.3. Smart Homes

A Smart Home (SH) monitors and manages home electronics and appliances through an Internet connection [33]. These devices or social products include thermostats, cameras, voice assistants, lights, doorbells, household appliances [9,20,34,35], and provide homeowners information on how much energy they have used on certain equipment or systems [11]. Moreover, an SH collects and analyzes data about the home environment, communicates that data to users and service providers, and improves the ability to manage various domestic systems through social products [12].

In addition, Marikyan et al. [10] classified the SHs into four groups based on the technology services:

1. Surveillance home: Householders receive alerts about possible natural disasters or security interventions. Besides, the SH gathers data from the environment to detect burglary threats.
2. Assistive home: This home type promotes the householders’ well-being through action recognition. Hence, three types of services are offered:
  - Senior oriented
  - Child oriented
  - Overall health-oriented
3. Detection and multimedia home: This home type detects and collects information from videos and photos of householders’ daily lives.
4. Ecological awareness home: Householders monitor and control their energy supply against demand through special sensors and automatic monitoring systems. Thus, this home type promotes environmental sustainability.
  - Smart Home Energy Management Systems have advanced IoT devices to convert a traditional home into an energy-aware home to reduce energy consumption and promote money savings [36].

**Figure 1:**
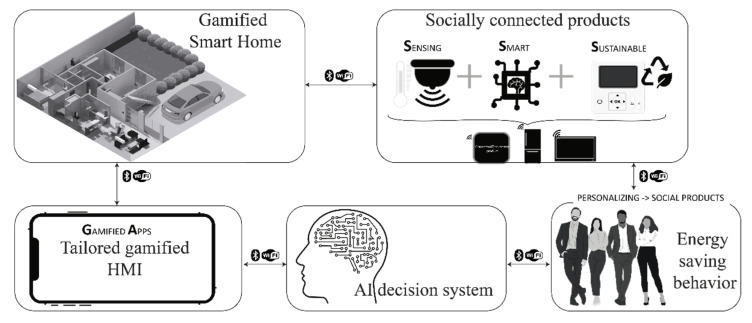
Gamified Smart Home structure.

However, a fifth type of home should be added, the Gamified SH. This type of home uses socially connected products [20,21,35,37,38,39,40,41,42,43] that profile end-users based on their personality traits, type of gamified user, and energy user [9,34,44,45] to propose tailored interfaces that help them understand the benefits of becoming pro-environmental or energy-aware. Figure 1 depicts an example of this type of home.

#### Fuzzy Logic and Smart Homes for Detection of Depressive Disorders

The computer scientist, Lotfi Zadeh from the University of California Berkeley, proposed in 1965 the fuzzy set theory as a class of sets based on membership grades from 0 to 1 [46]. The fuzzy set has inference rules that do not require a mathematical model of the real system but rather rules generated by experts, polls, or consensus-building [47,48]. Hence, fuzzy logic (FL) was created to model uncertainty based on linguistic words and sentences, known as linguistic variables, to associate them with human logic rather than the use of numerical values. The process begins with the fuzzification step that classifies in fuzzy sets the variables with an uncertainty metalinguistic degree. Following, the experts propose the linguist inference rules with an antecedent IF and a consequent THEN. Finalizing with the defuzzification step is the final process that determines the output values through fuzzy inference methods such as the Mamdani and the Sugeno inference [48].

Multi-sensor data fusion is a framework that collects information from multiple sensors to make inferences and create functions that compensate, process information, communicate, and integrate to provide quantitative measurements consistently [20,42,47]. The multi-sensor data fusion bases its concept on human beings’ and animals’ fundamental tasks, using multiple sensors to survive and track possible threats. Furthermore, this system relies on the decision-making stage because the information collected from the sensors requires a decision based on the sensed data.

Wlodarczyk et al. [49] proposed SWRL-F as an FL extension of the Semantic Web Rule Language because no precise information can be used. Hence, the knowledge representation in SWRL was simplified. Thus, their SWRL-F ontology constructed fuzzy rules in SWRL using Web Ontology Language. Thakur et al. [50] proposed a fuzzy inference system for diagnosing depression through the PHQ-9 to get the correct level of severity of depression among patients. They collected data from 50 patients from hospitals in Bangladesh. Leon et al. [51] proposed the SENTIENT project to identify early signs of depressive disorders in senior householders to ease affective support and care. A systematic review concluded that SHs influence senior users’ QoL, but low technology readiness requires diversification [52]. VandeWeerd et al. [53] deployed the HomeSense project in the community of older adults in The Villages, Florida. This ambient health and wellness platform monitors the age of senior people to recognize any relevant event that may affect their QoL. In [20], they used a voice assistant and cameras to track the householders’ daily mood to improve their QoL by promoting social inclusion and physical exercise. The multi-sensor system was used within the SH to identify the senior householders’ emotions.

The depression disorders at SHs are currently oriented to the senior householders [20,39,50,51,52,53]; however, depression affects all of us [1,2]. Hence, experts need to diagnose the depression disorder; questionnaires as the PHQ-9 help spot any depressive symptomatology as a pre-diagnosis.

Therefore, using artificial intelligence (AI) decision systems like adaptive neuro-fuzzy inference systems (ANFIS), fuzzy logic, or neural networks can provide reliable information about end-users behavior because AI techniques emulate human making decisions [47,54]. In addition, AI platforms have been used to ease conversation and social support within communities to assess digital health interventions [55,56]. Thus, it is possible to think of an integrated and complex system that monitors householders’ mental health to help as a pre-diagnostic depression symptom and send that information to the clinicians.

### 2.4. Gamification for Treatment Depression

Gamification uses game elements and game-design techniques in real context environments [57]. In [58], they performed a systematic review to identify how gamification and serious games were applied to support the treatment of depression. The gamification elements included scores, goals, and progress levels. For example, Lukas et al. [59], proposed a gamified smartphone-based intervention to reduce depressive symptoms. Thus, the gamification elements considered goals, progress levels, and a total number of views. Hungerbuehler et al. [60], considered story and feedback gamification elements for their chatbot proposal.

The Octalysis framework [57] helps as a guideline to develop gamified applications through two types of motivations to fulfill specific activities, for instance, avoid householders’ depression at home. Therefore, the communication between interfaces and the end-user is from a tailored human machine interface (HMI) within a gamification structure that includes feedback and adjustments based on the user’s level of depression to teach, motivate, and engage them to perform specific goals that avoid isolation or depression. Hence, Table 2 lists the two types of gamification structures used in this research. In extrinsic motivation, people are motivated because they want something they cannot get, and acquiring it infers outer recognition. For intrinsic motivation, the activity is rewarding by itself.

Consequently, to the best of the authors’ knowledge, proposals consider the householders to monitor any depression symptomatology at SHs using the PHQ-9, either through voice assistants or tailored human–machine interfaces (HMIs), has not been proposed.

Therefore, the research question proposed in this research was:

- What characteristics should an HMI framework consider helping the healthcare workers pre-diagnose depression using SHs as enablers?

To answer the research question, this paper proposes a three-step framework that assesses the nine questions to the end-user through ALEXA or a gamified HMI. Then, a fuzzy logic decision system considers three actions based on the nine responses: continue monitoring through Alexa and the HMI, suggest specialist referral, and mandatory specialist referral. These actions can only be seen by the healthcare system and/or to the end user’s emergency contact. Besides, a statistical analysis was performed on the NHANES dataset [30,31] to get the depression severity by age group as a guideline for the specialist to know how the householder behaves compared to the national dataset; in other words, if the householder has an expected behavior, is outside the expected behavior, or has an abnormal behavior compared with the NHANES dataset.

## 3. Material and Methods

The NHANES dataset that had the (PHQ-9) answers [31,32] were melted with the Demographic Variables and Sample Weights (P_DEMO) [61,62] to get the gender and age of each observation and perform further statistical analysis. Then, the SEQN or ID from the P_DEMO was compared to add the three additional variables to the PHQ-9 dataset. Thus, the melted dataset had 8965 observations and 13 variables; the last variable was the total score of the nine questions. Any observations with NA and the tenth question (DPQ100) were deleted as the DPQ100 required a healthcare professional to answer. The scope of this research is related to the depression symptomatology pre-diagnosis. Therefore, the cleaned dataset had 7882 observations. Table 3 depicts the code and description of each variable from the melted dataset.

The software employed during this research were:

- The statistical analysis and data cleaning considered the SPSS v. 25 and R-studio v. 1.4.1106.
- The fuzzy logic decision systems used LabVIEW v. 20.0.1.

Thus, the statistical analysis considered the cleaned dataset and consisted in:

- Demographic characteristics of the sample (mean age, male and female proportion, and median PHQ9 scores), for normally distributed data, the mean and standard deviation were reported. The median and interquartile range were reported for non-normally distributed data.
- Setting the normal, expected, and abnormal depression thresholds for each age group, by obtaining the 3, 25, 50, 75, and 97 percentiles.
- The associations between age group and depression severity and sex and depression severity were determined. These associations were achieved with a frequency table and a chi-squared analysis; if the chi-squared analysis was found significant, a Crammer’s V was performed to obtain the strength of the association. Furthermore, a Spearman’s correlation test was performed between age and PHQ9 score.
- Items 1, 2, and 9 are crucial, because according to DSM, to make a depression diagnosis, 4 of the nine questions have to be positive, and at least one of the five must be items 1 or 2 [25,28]. Moreover, if item 9 is positive, the psychological referral is mandatory. In order to assess the distribution and association of these items in the different age groups and sex, frequency tables and chi-squared tests were performed with Crammer’s V test whenever it applied.
- *p*-values below 0.05 were considered statistically significant.
- Cramer’s V results were interpreted as follows: 0.0–0.1 negligible; 0.1–0.2 weak; 0.2–0.4 moderate; 0.4–0.6 relatively strong; 0.6–0.8 strong; 0.8–1.0 very strong [63].

### 3.1. Fuzzy Logic Decision System

**Figure 2:**
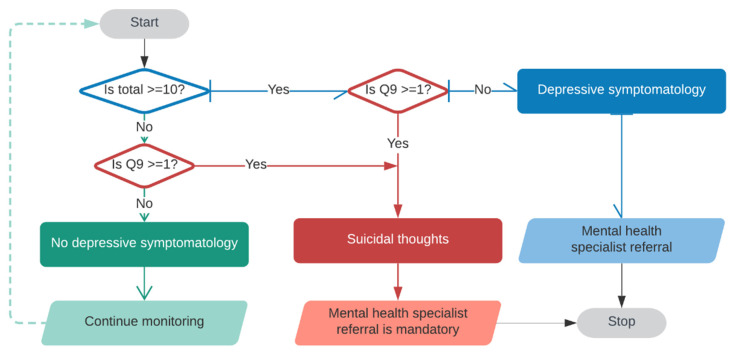
PHQ-9 algorithm for the fuzzy logic decision system.

Figure 2 shows the PHQ-9 algorithm used for the fuzzy logic decision system. This algorithm considers that if the nine answers had a score of less than ten and item nine had a “not at all” response, the action is to continue monitoring. On the contrary, if the score was greater than or equal to 10 and the ninth item was zero, the action is to consider depressive symptomatology. Nevertheless, if the ninth answer was greater or equal to 1, it is mandatory.

Two fuzzy logic decision systems were proposed. The first fuzzy logic decision system examined item 9 and the total score in the input system; the output system reflected the decisions explained in Figure 2: Continue monitoring, mental health specialist referral, and mental health specialist referral is mandatory. This decision system had 20 rules.

The second FL decision system considered the percentile score divided by age group starting with 20 to 29 years old, 30 to 39 years old, 40 to 49 years old, 50 to 59 years old, 60 to 69 years old, and ending with 70 to 80 years old age group for the input system. The output system was the same as the first FL decision system. This decision system had 18 rules. The fuzzy logic decision system was modeled in LabVIEW v. 20.0.1.

### 3.2. Human Machine Interface

In [21], some of the authors of this research proposed SH gamified HMIs for energy reductions. Hence, based on the premise of considering socially connected products at SHs such as connected thermostats, smart refrigerators, smart lightings, or smart TVs [9,38], a novel interface was proposed to take advantage of this type of HMIs to survey the householders the nine questions from the PHQ-9. Hence, the householders would not feel obligated to answer, and they could answer the survey if they want to, or even request Alexa to ask the questions instead of reading them.

**Figure 3:**
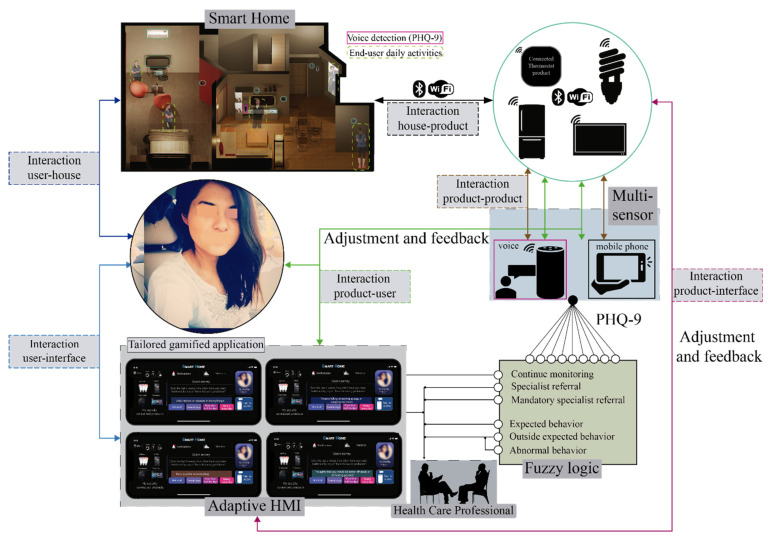
Smart Home Structure considering the PHQ-9 and the inclusion of Alexa.

Figure 3 depicts the interaction in the SH to monitor householders and run the pre-diagnosis PHQ-9. There are six types of interactions involved in this diagram: user-house, house-product, product-product, product-user, product-interface, and user-interface. This diagram reflects how the end-user is continuously in touch with the household appliances and elements. Thus, the interaction between the house and the product knows the profile usage at home, besides these appliances can be linked with the voice assistant as Alexa or the mobile phone to provide the interaction between products. Therefore, through the multi-sensor system, Alexa and the HMI can communicate with the end-user providing this interaction of product-user. The relevant part of this structure is the product–user interaction because the fuzzy logic decision system provides the actions required to pre-diagnose any depressive symptoms and, therefore, request a health care specialist.

## 4. Proposed Framework

**Figure 4:**
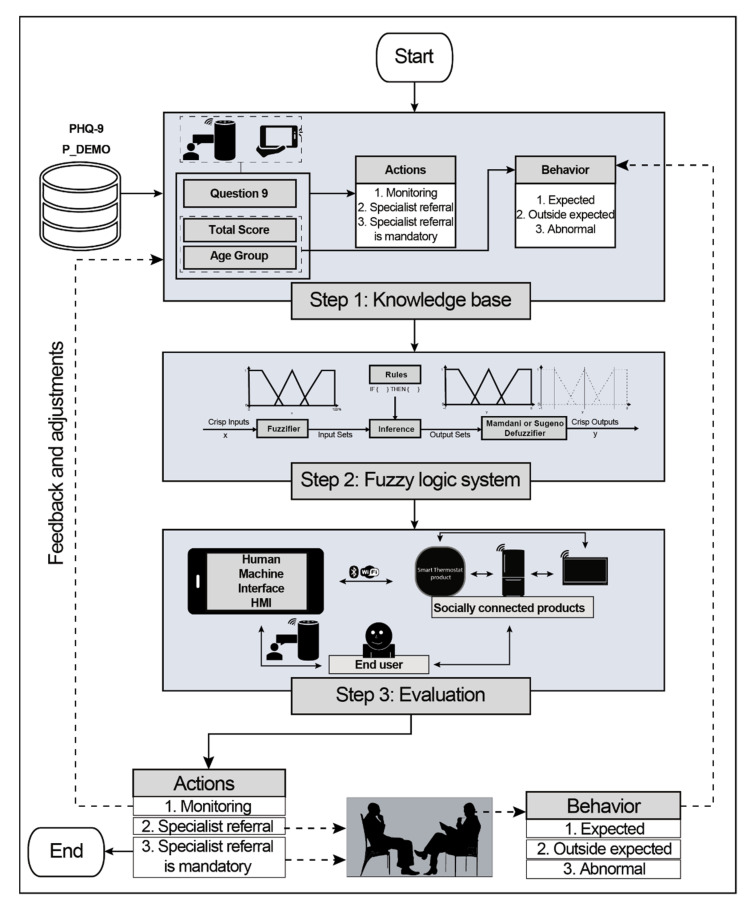
Proposed Framework.

Figure 4 shows the proposed framework for the depressive pre-diagnosis in the SH environment. This framework has three steps:

Knowledge base step: During this step, the NHANES dataset and P_DEMO were collected. The statistical analysis was performed to understand the depressive symptomatology better and take three actions based on the total score and question 9. Besides, the expected behavior by age group was proposed, so the health care professional can quickly review if the householder’s answers are with the national database or if the householder is behaving unexpectedly. In addition, the gamified elements for depression depicted in Table 2 were analyzed to propose them on the HMI.

Fuzzy logic step: This step has two parts. The first part considers question 9 and the total score to display which three actions were required to consider. The second part uses the age and the age group to display the type of behavior based on the three actions explained in the previous section.

Evaluation step: This step evaluates the interaction between the user and the household appliances to provide the interaction between product–product, product–user, and product–interface to help the health care professional better understand how the householder is behaving regarding the depressive symptomatology. This step provides continuous feedback to the user and the knowledge base to continue monitoring the end-user and pre-diagnose any householder’s depression.

## 5. Results

This section provides the results of each step and the HMI proposal in an SH Context. First, the knowledge base step provides the statistical analysis performed in the PHQ-9 and P_DEMO datasets. Then, the fuzzy logic decision system step provided the two types of decision systems and proposed rules for the depression pre-diagnosis. During this step, a LabVIEW program was suggested to interact with the ALEXA or HMI PHQ-9 questionnaire and the actions required based on the input values. Then, the evaluation step showed the results of the LabVIEW front panel and its connection to the HMI into an SH environment.

### 5.1. Knowledge Base Step: Statistical Analysis

The melted and cleaned database from NHANES PHQ-9 [31,32] and P_DEMO [61,62] containing 7882 observations were explored to obtain the main characteristics of the sample. The sample included 3865 (49%) males and 4017 (51%) females, with a mean age of 50.73 (s.d.: 17.42) and a median PHQ-9 score of 2 (IQR: 0–5). The division of PHQ-9 scores by age group reported a similar distribution with a median of 2, IQR of 0–5 or 0–6, third percentile of 0, and 97th percentile of 14, 15, or 16 (See Table 4).

Table 5 shows the depression severity by gender. The analysis reported a higher prevalence of depressive symptoms in women. A chi-squared test was performed to assess the association between gender and depression severity, and a weak association was found (*p*-value = 1.46 × 10^−17^; Crammer’s V = 0.104).

In depression severity by age group, a spearman correlation was performed using the age and PHQ-9 scores. No correlation was found between both variables (*p*-value = 0.000038; Rho = −0.046). A chi-squared test was performed to assess the association between age group and presence of depression, and a weak association was found (*p*-value = 1.23 × 10^−68^; Crammer’s V = 0.197) (See Table 6).

Due to the importance of item 9 in the questionnaire, since it assesses suicidal or self-harm thoughts, the distribution on its presence was explored by sex, age group, and depression severity. No association was found between sex or age group and suicidal thoughts in this sample (*p*-value = 0.545; *p*-value = 0.226, respectively); however, a relatively strong association was found between depression severity and presence of suicidal or self-harm thoughts (*p*-value = 0; Crammer’s V = 0.476).

Items 1 and 2 are crucial, because according to DSM, to make a depression diagnosis, 4 of the 9 questions must be positive, and at least one of the 5 must be items 1 or 2. These two items include depressive mood and reduced pleasure. Talking about question 1, a negligible association was found between its presence and sex or age group (*p*-value = 0.012, Crammer’s V = 0.028; *p*-value = 0.000085, Crammer’s V = 0.058, respectively). In question 2, a negligible association was found between it being positive and sex (*p*-value = 0.000028, Crammer’s V = 0.047), and no association was found between it being positive and age group (*p*-value = 0.086).

### 5.2. Fuzzy Logic Decision System Step

**Figure 5:**
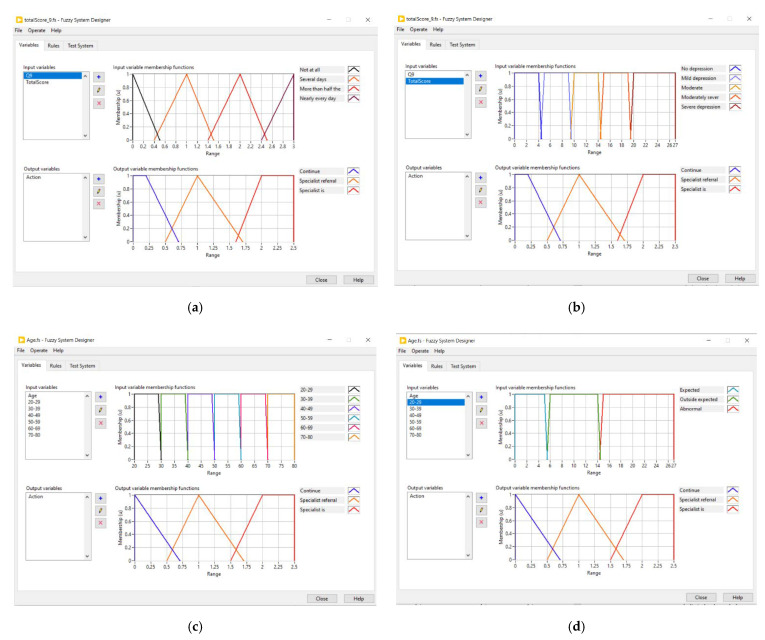
Fuzzy logic decision system: (**a**) first FL input and output variables: Q9; (**b**) first FL input and output variables: TotalScore.; (**c**) second FL input and output variables: age; (**d**) second FL input and output variables: age group.

Figure 5 shows the two types of FL decision systems created. Figure 5a shows the first FL decision system, the input and output rules used for Question 9. The input variables considered the four-scale answer depicted in Table 1 as the membership functions. The output variables were the three actions to perform depending on Question 9 and the total score. Figure 5b shows that the input variables considered the total score and the membership functions were the depression severity. Figure 5c shows the second FL decision system; the input variables considered for the age and the membership functions; Figure 5d shows the input variables considered by each age group and the type of behavior for the membership functions. In both Figure 5c,d, the output variables were the same as the first FL decision system. Besides, Table 7 shows the FL rules employed for the first decision system, and Table 8 depicts the FL rules for the second decision system.

**Figure 6:**
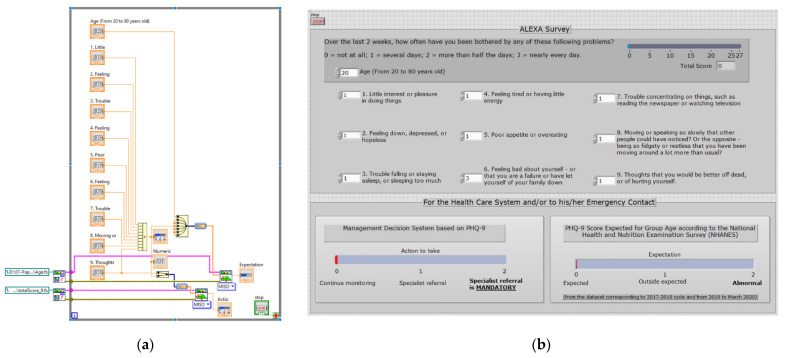
LabView project: (**a**) block diagram; (**b**) front panel.

Figure 6a depicts the block diagram used for the LabVIEW project. This diagram linked both FL decision systems to provide a single front panel. Figure 6b displays the front panel.

This proposal adds the nine questions and based on the last question and the total score, the actions are displayed on the lower part of the panel. Besides, considering the age and the total score, the expectation of depression by age group is displayed.

### 5.3. Evaluation Step

**Figure 7:**
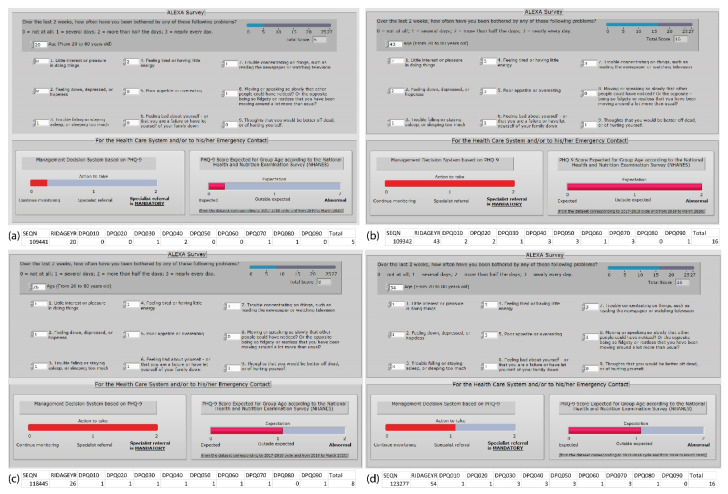
Dashboard results: (**a**) 20 years old individual with a total score of 5; (**b**) 43 years old individual with a total score of 16 and suicidal thoughts; (**c**) 26 years old individual with a total score of 8 and suicidal thoughts; (**d**) 54 years old user with a total score of 16.

Figure 7 shows some results on the LabVIEW front panel. The answers were based on the NHANES datasets. For instance, Figure 7a shows the answers received from SEQN 109441. This individual is 20 years old and had a score of 5, which is the same as mild depression; thus, the actions required for this individual are to continue monitoring, and this individual is under the expected national behavior. On the contrary, Figure 7b displays the SEQN 109342 for a 43-year-old individual with moderately severe depression. This individual answered, “several days” for the ninth question, and the system immediately moved toward the “health specialist referral is mandatory”. This individual behavior is not normal, and it is totally outside the expected behavior. Figure 7c shows an example for a 26-year-old individual with suicidal tendencies due to question 9; however, based on the total score and the age, this individual is outside the expected behavior. Finally, Figure 7d depicts the information for a 54-year-old user with a total score of 16 but with no suicidal thoughts; therefore, the action is to suggest a specialist referral but not in a mandatory manner; besides, this user is outside expected behavior but not abnormal behavior.

Furthermore, Table 9 exemplifies five of the 7882 observations, including the LabVIEW dashboard results. The full observations are available at [64].

### 5.4. Human Machine Interface in a Smart Home Context

**Figure 8:**
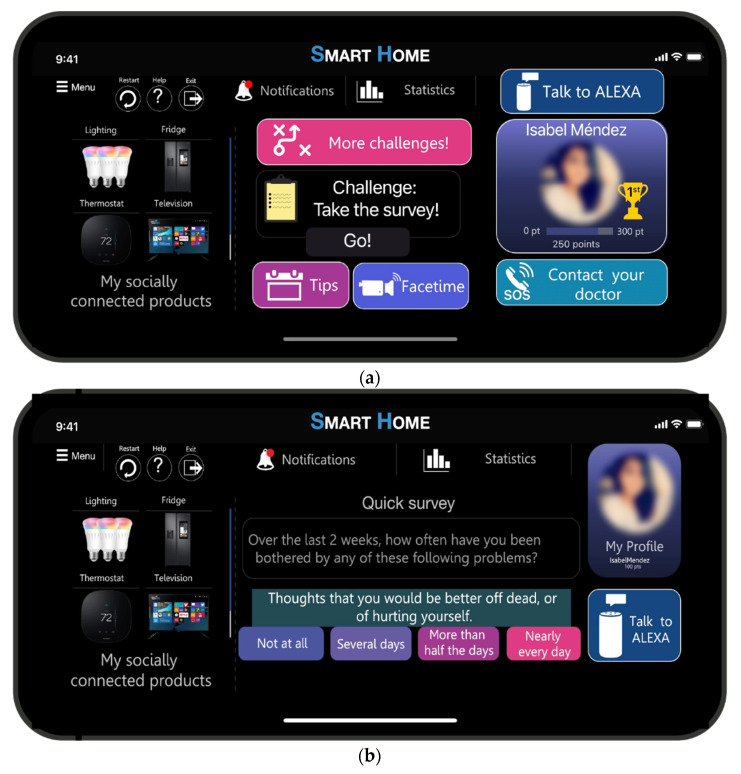
HMI oriented to pre-diagnosis using the PHQ-9: (**a**) Homepage of the gamified HMI; (**b**) Question 9 is depicted in the gamified HMI.

Figure 8 depicts the proposal of a gamified HMI in an SH context. This interface shows how to use interfaces that include connected products such as the thermostat, television, refrigeration, or lighting. This interface focuses mainly on the quick survey so the householder can answer either by clicking one of the four options of each question of the PHQ-9 or by selecting the option of “Talk to ALEXA”. Figure 8a shows the homepage, the extrinsic gamified elements that consider the challenges, the statistics, the picture profile, the points, and the leaderboard. On the contrary, the intrinsic gamified elements are related to the notifications, the tips, the choice perception by either talking to ALEXA or contacting the doctor, and by answering the survey. Figure 8b displays an example of this survey; thus, question 9 is asked. Therefore, once the householder answered the first question, the second question was displayed, and so on, until the tenth question. Hence, in both ways, the end-user can answer the questionnaire. However, this interface did not provide information to the householder regarding his/her mental health score. Therefore, the psychologist or psychiatrist needs to analyze the result to provide further actions to avoid any suicidal thoughts. Besides, this type of interface aims to engage the end-user in activities to achieve specific goals. For instance, this interface was connected to a thermostat where the householder interacted with the device and became energy aware [38].

## 6. Discussion

The characteristics that an HMI framework needs to consider to help the healthcare sector in the pre-diagnosis of depression using SHs as enablers are as follows:

1. A knowledge base gathers the population with depression symptomatology through available PHQ-9 answers to relate them with the proposal and identify if the householder is within the range of expected depression or requires more attention. During this step, the most common gamification elements for depression are collected.
2. A fuzzy logic step that helps as a decision system triggers three actions: continue monitoring, specialist referral, and specialist referral is mandatory. Furthermore, three expected behaviors are analyzed: expected, outside expected, or abnormal behavior.
3. The evaluation step assesses the interaction between the householder and depression by taking advantage of household appliances through gamified interfaces. The interface uses gamification elements to help as an enabler for helping in the pre-diagnosis of depression. The elements are divided into extrinsic and intrinsic. Moreover, this pre-diagnosis runs into a feedback and adjustment environment; hence, the householder receives points by answering the survey, contacting the doctor, and interacting with ALEXA or through video call. In [20], it is proposed to use video callings as social connectors to avoid social isolation or depression.

This proposal considered the NHANES PHQ-9 [31,32] and P_DEMO [61,62] datasets to build the three-step framework. For the first phase of the knowledge base, a statistical analysis was explored to obtain the main characteristics of the dataset and determine based on the age group the acceptable limits for expected behavior, outside behavior, and abnormal behavior of depression symptomatology. Then, the FL decision systems were built during the second step based on the statistical analysis and the algorithm depicted in Figure 2. Finally, the evaluation phase consisted in analyzing the database and comparing the results with the dashboard depicted in Figure 6. Besides, an HMI that considers the household appliances and takes advantage of the home page of this HMI is proposed. Hence, the HMI uses this home page of the SH interface to provide the PHQ-9 survey in two modalities: through the HMI or ALEXA. 

This paper proposes a novel approach by considering the SH environment and household devices to monitor the householder. This depressive symptomatology monitoring is directly proposed considering either the HMI questionnaire or through ALEXA. Due to the increase and acceptance of voice assistants, this option is preferred for individuals who feel more comfortable talking rather than interacting with mobile phones or tablets. This proposal is novel in terms of considering all the householders above 20 years old instead of just focusing on the senior users that the literature review had widely indicated [20,40,51,52,53,54]. Besides, depression is not exclusive to the older population [1]; depression can happen at any age, leading to poor QoL and affecting the economically active population [1,2]. The age range was considered for this proposal due to the sample analyzed in the PHQ-9 and P_DEMO datasets [31,32,61,62].

In addition, COVID-19 had accelerated anxiety, depression, and suicide due to economic and face-to-face restrictions. Therefore, this proposal aims to use smart homes as enablers for depression pre-diagnosis through the PHQ-9 assessment on HMI and help the health care system and professionals detect any symptoms of depression at home to improve the QoL of the householders.

However, this proposal has its limitation regarding which type of user could interact, for instance, with the HMI [5,7,8]. Consequently, research suggests that certain personality traits are open to using new gadgets or new technology as the openness or extraversion personality trait. However, for the neuroticism personality trait is complicated to accept new technology [34,44]. Moreover, to propose gamified interaction for depressed householders, further research is required to propose tailored activities that improve their mental health.

## 7. Conclusions

Employing fuzzy logic decision system allows the healthcare systems to monitor the householder’s health and avoid suicidal thoughts or depressive disorders. Furthermore, depending on the total score and the ninth item, this system compared the total score with the NHANES dataset to provide insights into the health care sector. Thus, it makes it easy to review if the householder is behaving according to the national metrics or requires further actions. Hence, this proposal is designed to be implemented in SHs by considering all end-users (non-typical and typical users). However, further research is needed to develop and fully test the interface, for instance, by enrolling a representative sample of subjects to evaluate using this HMI continuously.

This research aims to propose the integration of ALEXA and gamified HMI to assess the PHQ-9 and provide a pre-diagnosis of depression symptomatology to help the health care system and professionals detect any symptoms of depression at home. Besides, this proposal considers three actions based on the total score and the suicidal item: to continue monitoring through the HMI and ALEXA, health care specialist referral, and mandatory specialist referral. In addition to this integration, a statistical analysis was performed to provide the dashboard information that helps as a comparison for the specialist. For instance, the householder can have a score of 16 but can have an outside expected behavior or abnormal behavior. This conduct is related to the age group provided by the NHANES dataset. Another aspect to include in further research is to include, if possible, the use of household appliances and cameras to track householders’ activities and compare their activities with their responses. For instance, the householder could feel with poor appetite; however, the refrigerator could register and compare how many times the householder opened the refrigerator and check with the camera if the householder ate more or less than two weeks ago. Another example is checking if the individual is having trouble falling or staying asleep, or feeling tired. Besides, ALEXA or another voice assistant can track the householder’s voice to identify possible changes in their modulation if the householder is speaking slower or faster than a couple of weeks ago.

Although this proposal includes all types of users above 20 years old (non-typical and typical users), to validate this proposal meticulously in the real end-user market, these future actions are required:

- Evaluate and improve the proposed application with different scenarios and target populations. For instance, there are sectors with chronic diseases such as rheumatoid arthritis that commonly have depression. With this proposal, evaluate their performance through their treatment to analyze if this proposal helps in improving their depression symptomatology. Besides, the proposal should be evaluated outside controlled environments and in several countries in which any cultural factor needs to consider the framework.
- Update the framework and HMI by including SWRL technologies as they have more robust rules than FL. However, this research did not propose SWRL or SWRL-F because the objective was to generate a conventional knowledge base. Afterward, future work will explore the use of these rules, and their optimization will be evaluated.
- Submit this proposal into medical protocols to assess the system in a pilot study with real users.
- Include, if possible, the camera tracking to develop facial recognition and compare the answers with the camera feedback.
- Employ ALEXA in combination with cameras or other household appliances to analyze householders’ patterns and compare their behavioral patterns with their survey answers. For example, the perception of depression is important. Thus, if the end-user is feeling depressed, but the actions reflect the opposite, a message could be displayed through the gamified interfaces. Moreover, ALEXA could talk to the householders and explain how they have been acting to cheer them up.

## Figures and Tables

**Table 1:** Patient Health Questionnaire (PHQ-9).

Over the Last 2 Weeks, How Often Have You Been Bothered by Any of These Following Problems?	Not at All	Several Days	More than Half the Days	Nearly Every Day
1. Little interest or pleasure in doing things	0	1	2	3
2. Feeling down, depressed, or hopeless	0	1	2	3
3. Trouble falling or staying asleep, or sleeping too much	0	1	2	3
4. Feeling tired or having little energy	0	1	2	3
5. Poor appetite or overeating	0	1	2	3
6. Feeling bad about yourself—or that you are a failure or have let yourself of your family down	0	1	2	3
7. Trouble concentrating on things, such as reading the newspaper or watching television	0	1	2	3
8. Moving or speaking so slowly that other people could have noticed? Or the opposite—being so fidgety or restless that you have been moving around a lot more than usual?	0	1	2	3
9. Thoughts that you would be better off dead, or of hurting yourself.	0	1	2	3

**Table 2:** Extrinsic and intrinsic motivations used in gamification.

Extrinsic Motivation	Intrinsic Motivation
Challenges	Notifications
Levels	Messages
Dashboard	Tips
Statistics	Community
Profile picture or avatar	Collaboration
Points, badges, leaderboard	Competition

**Table 3:** Code and description of the melted dataset.

Code	Description
SEQN	Respondent sequence number.
RIAGENDR	Gender of the participant.
RIDAGEYR	Age in years of the participant at the time of screening. Individuals 80 and over were coded at 80 years of age.
DPQ010 to DPQ090	Represent each question described in Table 1.
Total	Total score up to 27.

**Table 4:** PHQ-9 percentile score divided by age group.

Score Percentile	Age Group					
	20–29	30–39	40–49	50–59	60–69	70–80
P3 (Expected low limit)	0	0	0	0	0	0
P25 (Expected)	0	0	0	0	0	0
P50 (Median)	2	2	2	2	2	2
P75 (Expected)	5	4	5	5	5	4
P97 (Expected high limit)	14	15	15	16	16	14.67

**Table 5:** Depression severity by gender.

Depression Severity	Male	Female
No	3033	2790
Mild	553	780
Moderate	180	296
Moderately severe	69	108
Severe	30	43

**Table 6:** Depression by age group.

Score Percentile	Age Group					
	20–29	30–39	40–49	50–59	60–69	70–80
No Depression	861	920	941	964	1143	994
Total Depression	336	289	869	397	402	316

**Table 7:** First FL decision system rules.

Rule	IF	AND	THEN
1	Q9 is “Not at all”	TotalScore is “No depression”	Action is “Continue monitoring”
2	Q9 is “Not at all”	TotalScore is “Mild depression”	Action is “Continue monitoring”
3	Q9 is “Not at all”	TotalScore is “Moderate depression”	Action is “Specialist referral”
4	Q9 is “Not at all”	TotalScore is “Moderately severe depression”	Action is “Specialist referral”
5	Q9 is “Not at all”	TotalScore is “Severe depression”	Action is “Specialist referral”
6	Q9 is “Several days”	TotalScore is “No depression”	Action is “Specialist referral is mandatory”
7	Q9 is “Several days”	TotalScore is “Mild depression”	Action is Specialist referral is mandatory
8	Q9 is “Several days”	TotalScore is “Moderate depression”	Action is Specialist referral is mandatory
9	Q9 is “Several days”	TotalScore is “Moderately severe depression”	Action is Specialist referral is mandatory
10	Q9 is “Several days”	TotalScore is “Severe depression”	Action is Specialist referral is mandatory
11	Q9 is “More than half the days”	TotalScore is “No depression”	Action is “Specialist referral is mandatory”
12	Q9 is “More than half the days”	TotalScore is “Mild depression”	Action is Specialist referral is mandatory
13	Q9 is “More than half the days”	TotalScore is “Moderate depression”	Action is Specialist referral is mandatory
14	Q9 is “More than half the days”	TotalScore is “Moderately severe depression”	Action is Specialist referral is mandatory
15	Q9 is “More than half the days”	TotalScore is “Severe depression”	Action is Specialist referral is mandatory
16	Q9 is “Nearly every day”	TotalScore is “No depression”	Action is “Specialist referral is mandatory”
17	Q9 is “Nearly every day”	TotalScore is “Mild depression”	Action is Specialist referral is mandatory
18	Q9 is “Nearly every day”	TotalScore is “Moderate depression”	Action is Specialist referral is mandatory
19	Q9 is “Nearly every day”	TotalScore is “Moderately severe depression”	Action is Specialist referral is mandatory
20	Q9 is “Nearly every day”	TotalScore is “Severe depression”	Action is “Specialist referral is mandatory

**Table 8:** Second FL decision system rules.

Rule	IF	AND	THEN
1	Age is “20–29”	20–29 is “Expected”	Action is “Continue monitoring”
2	Age is “20–29”	20–29 is “Outside expected”	Action is “Specialist referral”
3	Age is “20–29”	20–29 is “Abnormal”	Action is “Specialist referral is mandatory”
4	Age is “30–39”	30–39 is “Expected”	Action is “Continue monitoring”
5	Age is “30–39”	30–39 is “Outside expected”	Action is “Specialist referral”
6	Age is “30–39”	30–39 is “Abnormal”	Action is “Specialist referral is mandatory”
7	Age is “40–49”	40–49 is “Expected”	Action is “Continue monitoring”
8	Age is “40–49”	40–49 is “Outside expected”	Action is “Specialist referral”
9	Age is “40–49”	40–49 is “Abnormal”	Action is “Specialist referral is mandatory”
10	Age is “50–59”	50–59 is “Expected”	Action is “Continue monitoring”
11	Age is “50–59”	50–59 is “Outside expected”	Action is “Specialist referral”
12	Age is “50–59”	50–59 is “Abnormal”	Action is “Specialist referral is mandatory”
13	Age is “60–69”	60–69 is “Expected”	Action is “Continue monitoring”
14	Age is “60–69”	60–69 is “Outside expected”	Action is “Specialist referral”
15	Age is “60–69”	60–69 is “Abnormal”	Action is “Specialist referral is mandatory”
16	Age is “70–80”	70–80 is “Expected”	Action is “Continue monitoring”
17	Age is “70–80”	70–80 is “Outside expected”	Action is “Specialist referral”
18	Age is “70–80”	70–80 is “Abnormal”	Action is “Specialist referral is mandatory”

**Table 9:** Exemplification of the results, including the LabVIEW decision system. The complete table is reported in [64].

SEQN	RIDAGEYR	DPQ010	DPQ020	DPQ030	DPQ040	DPQ050	DPQ060	DPQ070	DPQ080	DPQ090	Total	Action to Take	Expectation
109266	29	0	0	0	0	0	0	0	0	0	0	Continue monitoring	Expected
119042	75	0	0	0	0	0	0	0	0	1	1	Specialist referral is MANDATORY	Expected
109994	73	3	3	3	3	2	0	0	3	0	17	Specialist referral	Abnormal
121279	28	1	1	2	1	2	1	1	0	0	9	Continue monitoring	Outside expected
124698	51	2	3	1	1	2	1	1	2	0	13	Specialist referral	Outside expected

## Data Availability

Data available in a publicly accessible repository. The data presented in this study are openly available in National Health and Nutrition Examination Survey P_DPQ_Dataset at https://wwwn.cdc.gov/Nchs/Nhanes/2017-2018/P_DPQ.XPT, and in National Health and Nutrition Examination Survey P_DEMO_Dataset at https://wwwn.cdc.gov/Nchs/Nhanes/2017-2018/P_DEMO.XPT. The results presented in this study are openly available in github at https://github.com/IsabelMendezG/PHQ9_FuzzyLogicResults.
